# Vector Sensor Steering-Dependent Performance in an Underwater Acoustic Communication Field Experiment

**DOI:** 10.3390/s22218332

**Published:** 2022-10-30

**Authors:** Fabricio A. Bozzi, Sérgio M. Jesus

**Affiliations:** Laboratory for Robotic Systems in Engineering and Science (LARSyS), University of Algarve, 8005-139 Faro, Portugal

**Keywords:** acoustic vector sensors, underwater acoustic communications, directional sensors

## Abstract

This paper shows the performance resulting from combining vector sensor directional components in an underwater acoustic communication experiment. The objective is to relate performance with transmission direction and range. Receiver structures based on beamforming and passive time-reversal are tested in order to quantify and compare the steerability impact of vector sensor directional components. A shallow water experiment is carried out with a bottom fixed two-axis pressure-gradient vector sensor. A ship suspended acoustic source transmits coherent modulated communication signals at various ranges and from several directions. Results show that one vector sensor can provide an up to 10 times smaller error bit rate than a pressure sensor, favoring communication robustness without size penalty.

## 1. Introduction

Reliable underwater acoustic communications (UWAC) provide convenient support for untethered submerged platforms for ranges beyond, say, 100 m [1]. Fixed or mobile submerged platforms need to communicate either to other submerged platforms or to a surface station. Mobile platforms, e.g., autonomous underwater vehicles (AUVs) and gliders, are particularly challenging for UWAC because acoustic communication channels are continuously varying due to changing spatial characteristics of the propagation media [2]. For those mobile assets, long-range communication allows guidance, status updates, and the sharing of on-the-fly sensor information [1,3]. Mobile platforms are also restricted in size, weight, and energy consumption, which explains why not many of those employ acoustic communications on board and, when they do, these are based on very simple low-power single transducer modems [4]. Typically, such acoustic transducers have an omnidirectional or a fixed directional pattern, from which this study differs, by using higher-order sensors, known as acoustic vector sensors.

Acoustic vector sensors are devices that separately measure pressure and directional components [5]. Vector sensors have been widely used for sonar applications to mitigate left-right ambiguity of line arrays and provide a directional gain even for low-frequency signals (under 300 Hz) [6]. It is notorious and expected that most of the literature on vector sensors addresses the direction-finding issues since a compact collocated device can improve the gain of an ordinary pressure sensor or enhance the gain of a pressure array [7,8,9,10]. Much work has been carried out regarding direction of arrival (DoA) estimation using vector sensors, either by additive or multiplicative channel combining methods [7,11,12,13], exploiting signal and noise subspace domains [14], higher-order array manifold [15], or artificial intelligence [16]. Although researchers have deeply investigated DoA methods, the use of this information for UWAC is still little explored.

For UWAC, vector sensors are relatively new, and their components are used as a single-input multiple-output system, acting itself as an equalizer [17]. Usually, the components are considered independent channels, and a matched-filter combining is used and implemented as versions of multichannel equalizers [17,18] or passive-time reversal (PTR) [19,20]. In such approaches, the advantage of the vector sensor over the pressure sensor is explained by its intrinsic directionality, both by improving signal-to-noise ratio (SNR) when the horizontal component is used, and by exploring channel diversity, which, in theory, could be provided by the vertical component in far-field scenarios.

Besides matched-filter methods, an investigation of beamforming for communication has shown that the provided SNR gain of a two-axis vector sensor may reduce bit error rate (BER) [21]. However, data analysis is reduced, and the presented result is limited to one fixed source-receiver range and direction. A recent study compared a beamforming approach using three-axis vector sensors to an adapted receiver structure that joins beam steering and passive-time reversal methods [22]. Although the results show the benefits of the proposed receiver structure by reducing the bit error and increasing robustness along range, the steerability is not deeply investigated since more transmission directions in different geographic quadrants are necessary for a complete and secure analysis.

Thus, this work intends to show, experimentally, the steerability impact of vector sensors on communications using a recent dataset of a shallow-water field experiment. Conducted under the European Multidisciplinary Seafloor Observatory Portugal (EMSO-PT) project in November 2021, this experiment (EMSO’21) allows us to test a point-to-point communication link between a surface platform and a bottom receiver. The receiver is a single two-axis vector sensor whose outputs were acquired by a newly designed recorder. During EMSO’21, the vector sensor was fixed to a tripod on the seafloor, and an acoustic source was suspended from a ship transmitting coded sequences at various ranges and from several directions around the vector sensor. One can notice that such geometry can be similar to an AUV operating close to the bottom with an onboard vector sensor and a surface transmitting station. The communication performance was quantified and compared using vector sensor individual components, a vector sensor beam steering (vs-bs), passive time-reversal (vs-ptr), and the joint beam steering and passive time-reversal (vs-bsptr) methods. Moreover, this work addresses the necessity of a preprocessing stage for particle velocity estimation when using a pressure-gradient vector sensor. Such a relevant analysis is complemented by the impact of DoA fluctuation in the BER and how directional ambiguities may influence communication performance. This study shows a clear relationship between communication performance, range, and source direction. The steerability of vector sensors is shown as an advantage, where the combining approaches constantly outperform the omnidirectional pressure sensor.

This paper is organized as follows: analytical expression of pressure-gradient vector sensors and the tested receiver structures are shown in Section 2; the EMSO’21 experiment is described in Section 3; the experiment’s results are presented in Section 4; Section 5 presents analysis and discussion; and finally, Section 6 presents the conclusion.

## 2. Vector Sensor and Underwater Acoustic Communications

### 2.1. Pressure-Gradient Vector Sensor

An acoustic vector sensor refers to a generic device that measures the vector properties of the acoustic field. Since we refer to a vector measurement, amplitudes relative to directions are the directional outputs. The intrinsic directionality of vector sensors can be obtained by pressure-gradient or particle velocity [8]; this relation can be seen through the Euler’s equation:

(1) $$v=-\frac{1}{j\omega \rho_{0}}\nabla p,$$

where *v* is the particle velocity, ∇ is the gradient operator, *p* is the pressure, ω is the angular frequency, and $\rho_{0}$ is the medium static density. Assuming plane-wave condition, $p_{v}=-\rho_{0}cv$, where *c* is the sound speed and $p_{v}$ is the so-called pressure-equivalent particle velocity. Thus, Equation (1) becomes:

(2) $$p_{v}=\frac{1}{jk}\nabla p,$$

where k=ω/c=2π/λ is the wavenumber, λ being the wavelength. In the present work, we use a pressure-gradient vector sensor where the directional information (for each axis) is estimated using a pair of hydrophones. Consider two closely-spaced, identical hydrophones receiving an acoustic wave from direction θ. The output difference of these two hydrophones is given as [11]:

(3) $$\Delta p=j2p_{0}\sin\left(\frac{k^{\prime }s^{\prime }\cos\theta }{2}\right)\approx jp_{0}k^{\prime }s^{\prime }\cos\theta ,\;∵s\ll \lambda$$

where $p_{0}$ is a pressure reference, θ is the angle between the propagation direction and the pressure sensors axis, *s* is the distance between hydrophones, and the superscript ${\left[\right]}^{\prime }$ is used to represent an unknown parameter. Although the simplification in Equation (3) is restricted to s≪λ, in practice, technological limitations, such as the size of hydrophones or electronic parts, may not allow such theoretical consideration. Thus, as stated in [11], a spacing from λ/4 or less may be sufficient for such a requirement. Considering the first-order approximation $\frac{\Delta p}{s}=\frac{\partial p}{\partial s}$ and replacing Equation (3) in Equation (2) gives:

(4) $$p_{v}=\frac{1}{jk}\frac{\Delta p}{s}=\frac{1}{jks}jp_{0}k^{\prime }s^{\prime }\cos\theta ,$$

where if $k^{\prime }=k$, $s^{\prime }=s$, and the plane-wave assumption is valid for the operational frequency range, then Equation (4) becomes:

(5) $$p_{v}=p_{0}\cos\theta .$$

The cosine in Equation (5) shows the intrinsic directional nature of a vector sensor component. Additionally, the differential output of Equation (3) is proportional to ks, which makes it frequency- and spacing-dependent, a limitation regarding the necessary dynamic range [8]. On the other hand, its low sensitivity to non-acoustic interference (e.g., movement, turbulence, or vibration) makes it better suited than accelerometer-based vector sensors onboard moving platforms. The vector sensor output given in Equation (3) presents a dipole ambiguity, which may be a drawback for direction estimation purposes. Thus, an additional hydrophone is used as a reference, usually positioned at the geometric center of the hydrophone pair, resulting in a (1+δcosθ) term, which mitigates the dipole ambiguity according to a design factor δ [11].

### 2.2. Tested Receiver Structures

The communication receiver structures employ three stages: Doppler compensation, individual components or a channel combining method, and an equalizer. Three vector sensors channel combining methods are used: beam steering (vs-bs); passive time-reversal (vs-ptr); and joint beam steering and passive time-reversal (vs-bsptr). The Doppler compensation is based on the ambiguity function with a block compensation, where the input signals are resampled and used individually or combined [23].

In the beam steering method, the output is given by [11]:

(6) $$y_{bs}=r_{p}+\delta \left[r_{vx}\cos\theta_{0}+r_{vy}\sin\theta_{0}\right],$$

where $r_{p}$, $r_{vx}$, and $r_{vy}$ are the pressure and pressure-equivalent particle velocity components (Doppler compensated). δ is a design factor adjusted according to a desired beam response, and $\theta_{0}$ is a chosen steering angle, where we may set a $\theta_{0}$ value ($-\pi \leq \theta_{0}\leq \pi$) or use an estimated angle ${\hat{\theta }}_{0}$ from a DoA estimation. There are several DoA estimators available in the literature but here, the Bartlett estimator is used, where the beam response in the *f* single frequency is given as [24]:

(7) $$B\left(f,\delta ,\theta \right)={\left[1\;\cos\theta \;\sin\theta \right]}^{H}\;C\left(f,\delta \right)\;\left[1\;\cos\theta \;\sin\theta \right],$$

where $C\left(f,\delta \right)=\frac{1}{N}\sum_{f-\Delta f/2}^{f+\Delta f/2}{\left[R_{p}\;\delta R_{vx}\;\delta R_{vy}\right]}^{H}\left[R_{p}\;\delta R_{vx}\;\delta R_{vy}\right]$ is the sample cross-correlation matrix estimated in the frequency domain, in the Δf bandwidth with *N* samples, and R≡R(f). The estimated azimuth source direction angle is given by:

(8) $${\hat{\theta }}_{0}\left(f,\delta \right)=arg\max_{\theta }B\left(f,\delta ,\theta \right).$$

One can notice that the vector sensor beam steering combines the directional components with the pressure, which leads to an ambiguity mitigation benefit [11]. Another advantage is that the beam steering produces one output, demanding only a single channel equalizer.

In the passive time-reversal method, the m-th matched-filter output is given by [25]:

(9) $$y_{m}\left(t\right)={\hat{h}}_{m}^{*}\left(-t\right)r_{m}\left(t\right),$$

where ${\hat{h}}_{m}$ is the estimated pressure/particle velocity channel impulse response (CIR), and $r_{m}$ is the Doppler compensated pressure/particle velocity components. Although Equation (9) is used with the vector sensor directional components, it presents no difference from the standard PTR employed for pressure-only arrays. Thus, we may ask if the simple direct employment of this method for vector sensors is appropriate, taking into account, for instance, a scenario where the source transmits at one of the vector sensor axis directions. In this scenario, the orthogonal axis presents low SNR since the dipole patterns are orthogonal, and including such a noisy channel may not be advantageous. Therefore, in the joint beam steering and passive time-reversal method, a soft normalization is used and Equation (9) becomes:

(10) $$y_{m}\left(t\right)={\hat{h}}_{m}^{*}\left(-t\right)r_{m}\left(t\right)u_{m},$$

where $u_{1}=u_{p}=1$, $u_{2}=u_{vx}=\cos\left({\hat{\theta }}_{0}\right)$, and $u_{3}=u_{vy}=\sin\left({\hat{\theta }}_{0}\right)$. ${\hat{\theta }}_{0}$ is the estimated source direction from the beam steering stage, which weights the horizontal components emphasizing the component to source direction and attenuating a possible noisy component. Whereas in the vs-ptr, three outputs are produced for the vs-bsptr, and four outputs are used, one from Equation (6) and three from Equation (10).

The last stage of the receiver structures is a single decision feedback equalizer (DFE) for the vs-bs or a multichannel DFE for the vs-ptr and vs-bsptr. The DFE comprises M feed-forwards (according to the method) and a feedback filter. A second-order phase-locked loop (PLL) is embedded into the DFE to compensate for phase fluctuation. The adaptive cost function is performed using the recursive least square (RLS) algorithm [26].

## 3. EMSO’21 Experiment

**Figure 1:**
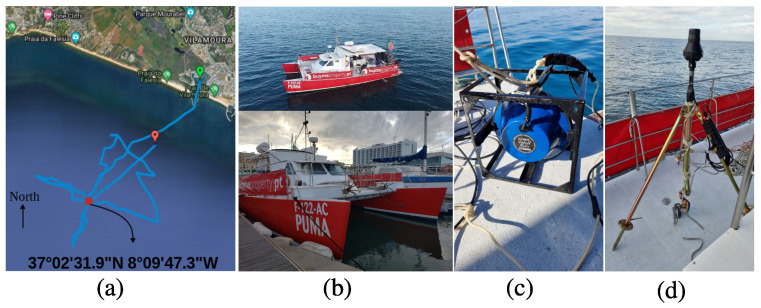
EMSO’21 experiment: satellite view with the ship sailing route and vector sensor positioning (red dot) (**a**); used ship (**b**); Lubell sound source (**c**); M35 vector sensor on top of the tripod, with the autonomous recorder at one of the tripod’s legs (**d**).

The EMSO’21 experiment took place off Vilamoura port, on the south coast of Portugal, on 24 November 2021. In this experiment, a point-to-point communication test was performed, where the receiver was a single vector sensor placed on the bottom, and the sound source was tied to a ship. Figure 1a shows the satellite view of the experiment area, highlighting the ship sailing route and the vector sensor positioning (red dot). The ship used in the test is shown in Figure 1b, which left the Vilamoura harbor at 9h00 (local) and went approximately 6 km in the southwest direction, where the vector sensor was deployed. Figure 1c shows the Lubell-916C sound source, which transmitted signals in the band from 3 to 13 kHz, where the transmit voltage response is approximately 154 dB/μPa/V (5 kHz) and 160 dB/μPa/V (10 kHz). The vector sensor was attached to the top of a tripod, and an autonomous recorder at one of the tripod’s legs, as shown in Figure 1d. A 10 kg weight was fixed at the tripod’s center bar in order to guarantee a deployment as vertical as possible and prevent roll-over due to sea currents.

The vector sensor is the two-axis pressure-gradient GeoSpectrum model M35, designed to operate from 100 Hz to 15 kHz, and which measures pressure and two orthogonal directional components (x-y components) [27]. The directional components’ axis references are considered true references, where the x-component is toward north and the y-component toward east. This axis compensation is performed by a clockwise rotation matrix using the north magnetic angle from an internal orientation sensor. The autonomous acquisition system was used to synchronously record the three vector sensor components (pressure, x, and y) at the sampling frequency of 39,062 Hz with a 24-bit of dynamic range.

**Figure 2:**
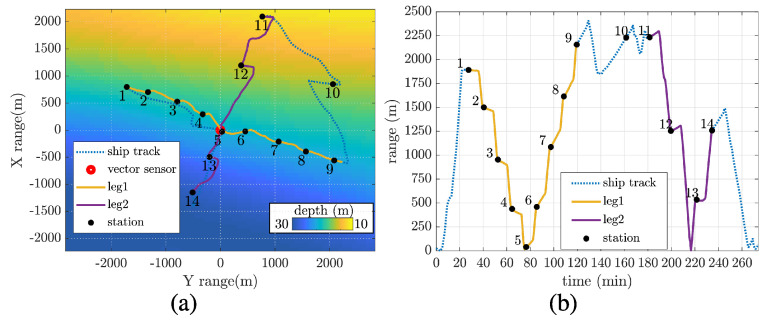
EMSO’21 experiment: ship track over area bathymetry in X–Y range centered at vector sensor/tripod position (37.04235° N, −8.16359° W), and transmitting stations (**a**); range between ship and vector sensor position along time (**b**).

Figure 2a shows the bathymetry in an X-Y range Cartesian plot with the vector sensor/tripod assembling position displayed at the origin (lat-lon 37.04235° N, −8.16359° W). The vector sensor was placed at approximately 2 m from the bottom, where the local water depth is 20 m. From the vector sensor deployment position, the ship goes along two tracks: leg1, which is an approximately 20 m depth isobathymetric transmission path, and leg2, which represents a downslope path. Notice that the figure’s axes were established according to the M35 reference (x is toward north and the y toward east, with azimuth angle θ clockwise). The planned transmission stations (black dots) 1, 2, up to 14, are considered fixed stations even if small displacements were noticed due to the ship drift. In Figure 2b, the ship-to-vector sensor range along time is shown, where the leg1, leg2, and the transmission stations are also highlighted. The communication signals transmitted at those stations were generated by the sound source suspended at approximately 7 m depth from a surface buoy tied at 3 m from the ship’s stern. This work analyzes a binary phase-shift keying (BPSK) communication signal at 2 kbits/s data rate and a carrier frequency of 5 kHz (effective 3 kHz bandwidth). Fifty packets of one second were transmitted, where each packet was a random series composed of 2000 symbols. A 255 symbol m-sequence preamble was used for synchronization and Doppler compensation. The signal was filtered by a root-raised-cosine pulse shape with a roll-off factor of 0.5.

## 4. Results

**Figure 3:**
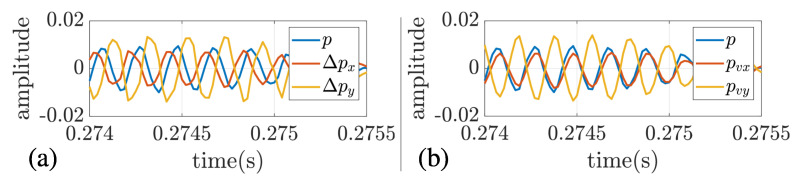
Received time series at station 3: pressure and pressure-difference channels (**a**); pressure and estimated pressure-equivalent particle velocity components (**b**).

The outputs of the vector sensor model M35 are pressure-difference Δp as shown in Equation (3), and the pressure-equivalent particle velocity components ($p_{vx}$ and $p_{vy}$) are estimated using Equation (4) with c=1516 m/s and s=0.05 m. A first check of the directional components (Δp or $p_{v}$) can be made by analyzing their phase, referenced to the omnidirectional hydrophone. An example can be seen in Figure 3, where the time series for pressure and the directional channels are shown for station 3. For Δp, a lead signal referenced to pressure represents north (for x-component) or east (for y-component), whereas a lag signal referenced to pressure represents south (for x-component) or west (for y-component). In Figure 3a, $\Delta p_{x}$ is lead, which represents north, whereas $\Delta p_{y}$ is lag, representing west. Thus, it is clear that the source direction comes from the northwest quadrant. For $p_{v}$, if pressure and particle velocity are in-phase, it represents a signal from the northeast quadrant and vice-versa. In Figure 3b, pressure and the estimated particle velocity $p_{vx}$ are in-phase, whereas pressure and $p_{vy}$ are in counter-phase. Thus, it represents a signal from the northwest quadrant as Δp. 

**Figure 4:**
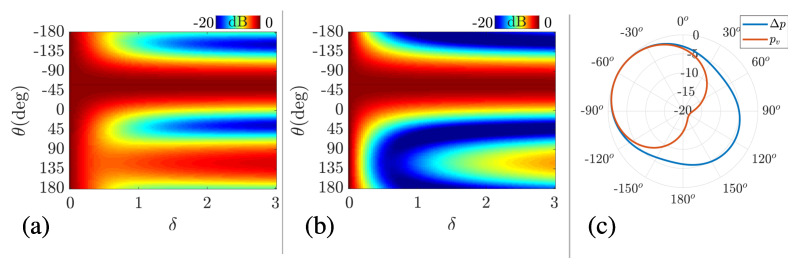
Energy detection for varying δ for station 3 using Δp (**a**) and $p_{v}$ (**b**). In (**c**), the beam response for Δp and $p_{v}$ is compared for δ=0.5.

The impact of the vector sensor channel combining communications can be associated with the spatial filtering capability, analyzed here by energy detection. The beam response for station 3 was analyzed by varying the δ factor, where it is known that the source is at approximately −60°. In Figure 4a, Δp is used, and the main lobe is noticed in the source direction, although ambiguity is verified as δ increases. In Figure 4b, the ambiguity is mitigated, and the maximum ratio between the main lobe and the sidelobe is found for δ=0.5. Figure 4c shows the beam response with δ=0.5 for both Δp and $p_{v}$, where the ambiguity mitigation is apparent, and the cardioid-like shaped response is obtained. Thus, one can conclude that the proper δ value is 0.5 for a backside ambiguity mitigation, which is set for quantifying the communication performance.

**Figure 5:**
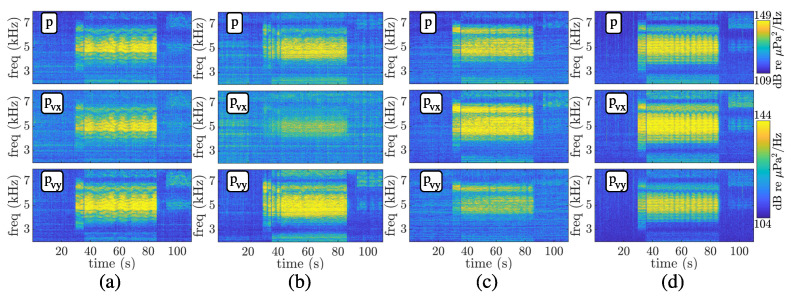
EMSO’21 spectrogram for pressure (*p*) and pressure-equivalent particle velocity ($p_{vx}$ and $p_{vy}$) during stations 3, 7, 12, and 14, from (**a**–**d**), respectively.

Spectrograms of the received communication signals for the pressure (*p*) and pressure-equivalent particle velocity ($p_{vx}$ and $p_{vy}$) for stations 3 (a), 7 (b), 12 (c), and 14 (d) are shown in Figure 5. The spectrograms show the BPSK communication signals that also contain a sequence of linear frequency modulation (chirp) as a probe for future use in automatic detection algorithms, not treated here. The same normalization was used in the spectrograms with the objective of showing attenuated/amplified components. One can relate the power spectrum amplitude of the components with the transmitting stations. For instance, in Figure 5b, $p_{vx}$ is highly attenuated since the sound source is transmitting at approximately 100°, whereas in Figure 5c, $p_{vy}$ is the attenuated component since the sound source is emitting from north (20°).

**Figure 6:**
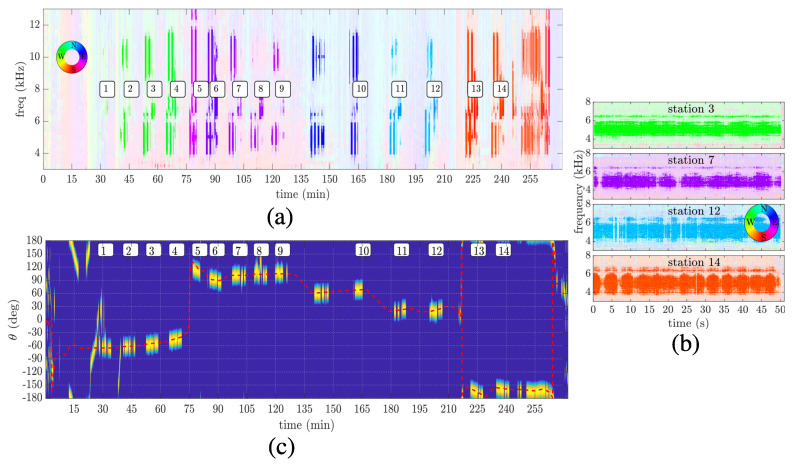
EMSO’21 azigram: during the overall communication test, where the number in boxes indicates transmitting stations (**a**); during transmissions at stations 3, 7, 12, and 14 (from top to bottom) (**b**); and energy detection using Bartlett estimator with estimated azimuth angle using GPS info (dashed red line) (**c**).

Figure 6 shows the azigram of recorded data during the overall communication test (a), during stations 3, 7, 12, and 14, from top to bottom (b), and the overall energy detection (c). An azigram is a condensed way to show the directional information over frequency and time. In fact, an azigram is analogous to a spectrogram, although the latter shows the power spectral density instead of the source direction estimation as a colormap. Interested readers can find a full study on azigrams in [28] and references therein. Here, the azigram was used as an analysis tool to check for transmitting directions and ambiguity issues. Figure 6a,b were obtained using the Bartlett estimator, where Equation (8) was used with a frequency step of 50 Hz, Δf=100 Hz, and an integration time of 0.34 s (a) and 0.08 s (b). Alpha transparency was used for clear visualization, considering the intensity of Equation (7). For comparison purposes, the broadband energy detection using the Bartlett estimator is shown in Figure 6c, where the estimated azimuth angle using GPS info is shown in the dashed red line. An empirical threshold of 80% is used to visualize transmission intervals better, although some fishing vessels are also detected up to minute 45. Moreover, it is possible to assimilate the transmitting stations with the source direction and frequency by comparing Figure 6c with the estimated directions in Figure 6a. Note that other communication signals were transmitted in different bands and modulations, not treated here. In Figure 6b, besides the directional information, a fading effect is observed within the fifty seconds of transmission. However, this figure clearly shows no directional ambiguity over the communication bandwidth. This fact is important since the azimuth angle, used for beam steering, is estimated using the full bandwidth, and this estimation could be impacted when some frequency presents ambiguity.

**Figure 7:**
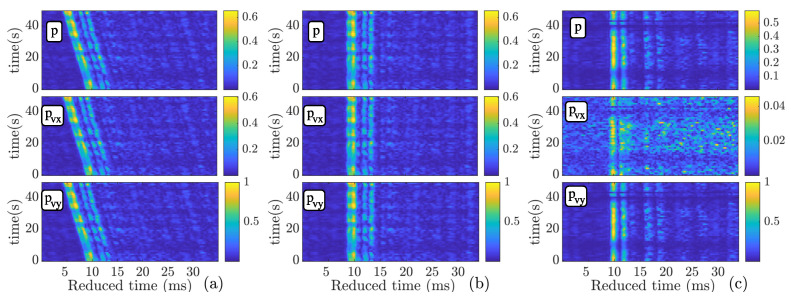
Estimated CIR for stations 3 (**a**,**b**) and 6 (**c**) for pressure (*p*) and pressure-equivalent particle velocity ($p_{vx}$ and $p_{vy}$) (from top to bottom).

Figure 7 shows the CIR of the vector sensor components for stations 3 and 6 for pressure (top), $p_{vx}$ (middle), and $p_{vy}$ (bottom) in a normalized scale. The CIR estimation is based on a correlation estimator, where the m-sequence was used as a replica. An alignment due to the Doppler was performed using the synchronization sample. In Figure 7a, the CIR of station 3 is not aligned, and the Doppler effect due to ship drift is noticed. Since the ship is moving toward the vector sensor (see Figure 2b), time compression is observed. Figure 7b shows the CIR of station 3, where the alignment is performed using the synchronization sample. In Figure 7c, the aligned CIR of station 6 is shown, where one can notice the amplitude difference between the components. The CIR of station 6 demonstrates the attenuation of $p_{vx}$, where the performance of methods that use such a noisy channel may be degraded.

**Figure 8:**
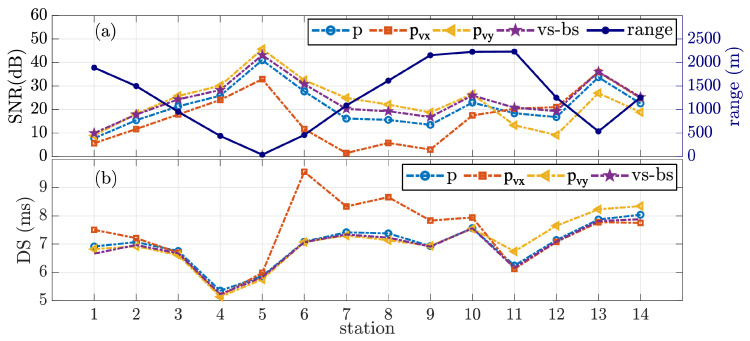
Estimated SNR (**a**) and RMS delay spread (**b**) along stations for pressure (*p*), directional components ($p_{vx}$ and $p_{vy}$), and for vector sensor beam steering method (vs-bs).

Figure 8a shows the estimated input SNR for pressure (*p*), directional components ($p_{vx}$ and $p_{vy}$), and vector sensor beam steering (vs-bs). The SNR for vs-bs was estimated using the steered output. The SNR for the passive time-reversal outputs is not shown since the combining gain is achieved after the equalizer. The SNR estimation was performed in the band from 4 to 6 kHz, and it uses a 50 s interval of the received signal and ambient noise starting 1 min before the received signal. For convenience, the vertical right-hand side dark-blue color axis shows the vector sensor to source range. The RMS delay spread (DS) is shown in Figure 8b, which characterizes the “severity” of the CIR, where a more extended time spread may represent a more severe channel [19]. The DS was calculated using the time-invariant CIR (power-delay profile [29]), where a −20 dB threshold was used. The DS for the passive time-reversal output is not shown since the resulting CIR was achieved after the equalization stage.

**Figure 9:**
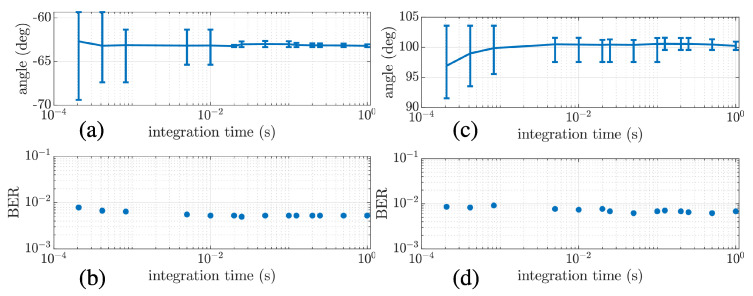
Estimated angle and BER varying the integration time for stations 2 (**a**,**b**) and 7 (**c**,**d**), respectively.

A relevant aspect to consider is the impact of angle fluctuation on the BER performance of vs-bs, which can be investigated using Figure 9. This figure shows the estimated angle and the BER according to the integration time for stations 2 (a,b) and 7 (c,d), respectively. One can note that the azimuth angle may fluctuate depending on the integration time used to estimate the correlation matrix. Discrete integration time values were used from 0.2 ms to 1 s, where 1 s is a full data packet interval. In Figure 9a,c, the error bar represents the mean value and the percentiles of the 50 packets. As expected, the fluctuation is higher as the integration time reduces. However, no relevant difference in the BER was noticed for the tested integration time, even for more than 10° of fluctuation. Thus, there is no advantage of using such a small integration time value since the error does not reduce. Furthermore, it is not practical to process in such a small interval (i.e., updating the DoA estimation), which motivates the use of a full data packet in this study.

**Figure 10:**
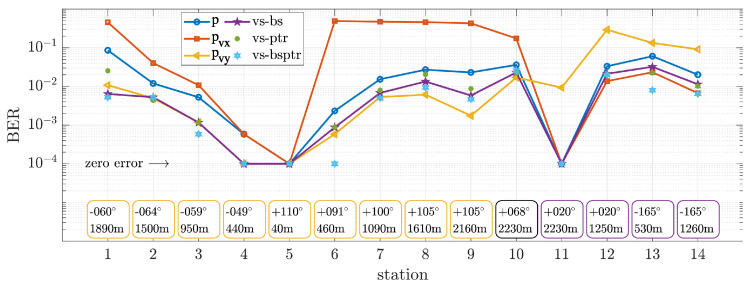
BER performance for pressure (*p*), directional components ($p_{vx}$ and $p_{vy}$), vector sensor beam steering (vs-bs), vector sensor passive time-reversal (vs-ptr), and the joint vector sensor beam steering and passive time-reversal (vs-bsptr). Bottom boxes display the source direction and range for convenience.

Figure 10 shows the BER performance for each station. The DFE uses 34 feed-forward and 20 feedback taps for adaptive fractionally spaced equalization with oversampling factor 2. The RLS forgetting factor is set empirically to 0.997. Moreover, the integral and proportional PLL coefficients are 0.01 and 0.0001, respectively. The fifty packets were treated as independent, and the BER was estimated as a median value. In Figure 10, the performance is quantified for vs-bs, vs-ptr, and vs-bsptr. For comparison purposes, the performance for *p*, $p_{vx}$, and $p_{vy}$ is also shown, separately. Note that bottom boxes show the source direction and range, highlighting leg1 and leg2 routes. Moreover, a zero error indicator shows the lower bound to account for errors that cannot be estimated with the limited number of samples available.

**Figure 11:**
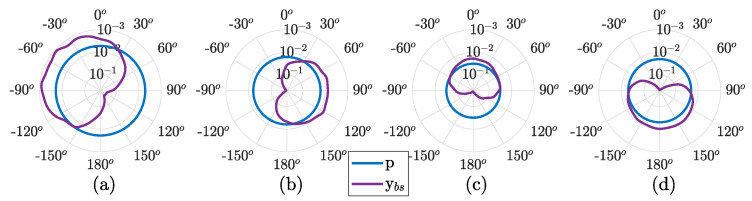
Polar BER for pressure (*p*) and beam steering (*y_bs_*) using Equation (6), for stations 3, 7, 12, and 14, from (**a**–**d**), respectively.

Figure 11 shows BER in polar plots using Equation (6) for discrete angles $-\pi \leq \theta_{0}\leq \pi$ in $y_{bs}$. Performance of the pressure sensor (*p*) is shown for reference. From (a) to (d), the BER performance is shown for stations 3, 7, 12, and 14, respectively. These transmitting stations were chosen for analysis since they are in different geographic quadrants, where steerability can be verified. Note that the polar graphs have a logarithm inverted axis, where the highest BER value (0.5) is at the center. The BER and beam pattern similarity can be observed using the axis in this format. 

## 5. Analysis and Discussion

### 5.1. Leg1 Sailing Route

For leg1, from stations 1 to 9, the source approaches the vector sensor from 1890 m range (station 1), passing at the closest range (40 m, station 5), and moves away up to 2160 m range (station 9). A direct relation between SNR and range is expected since the source level is kept constant throughout the experiment. The SNR curve for the pressure component of Figure 8a confirms this relation, where its maximum was obtained at the closest range. Regarding the SNR of directional components, it is essential to consider that the source follows a predominantly west–east path. Thus, a higher value is expected for $p_{vy}$ compared to the pressure, which is verified. Contrarily, a lower value for $p_{vx}$ than for the pressure is confirmed. A visual example of the SNR for each component is shown by the spectrograms of Figure 5b for station 7, where the power spectrum amplitude of $p_{vx}$ is strongly attenuated.

In Figure 8b, the DS values for *p*, $p_{vy}$, and beam steering (vs-bs) indicate similar channel complexity. The DS is about 7 ms long for pressure, $p_{vy}$, and vs-bs during leg1, except for stations 4 and 5. This result suggests that the equalizer deals with similar channel complexity, and SNR could be predominant for the achieved performance. The DS for $p_{vx}$ presents similar values to *p*, $p_{vy}$, and vs-bs, up to station 5. Then, the DS values for $p_{vx}$ are higher than *p*/$p_{vy}$/vs-bs from stations 6 to 9. These higher values are associated with imprecise CIR estimation due to the high attenuation of $p_{vx}$, as shown in Figure 7c for station 6. The DS for the beam steering presents similar values to *p* and $p_{vy}$’s DS, which is expected since these are the predominant components in the combination.

In Figure 10, the BER analysis for the pressure component shows a direct relationship between performance and range. The SNR impact is perceptible since the performance is inversely proportional to the SNR curve. However, the CIR variation among stations also affects the performance. For instance, stations 4 and 6 have similar ranges and also SNR but present different performances. In fact, the DS for these two stations indicates that the CIR of station 6 is more severe than that of station 4. For $p_{vy}$, the SNR gain of approximately 6 dB (±3 dB) relative to the pressure component reflects a BER performance improvement up to 10 times (station 1). Moreover, the performance for $p_{vx}$ is harshly degraded from stations 6 to 9. In these stations, the received signal is highly attenuated for $p_{vx}$ since the source direction is approximately +90°.

The vs-bs presents a similar BER performance to $p_{vy}$ in the leg1 sailing route. From stations 1 to 5, a performance better than or equal to that of $p_{vy}$ is noticed, with up to 10 times less error than the pressure component (station 1). However, from stations 6 to 9, $p_{vy}$ SNR is higher than the vs-bs SNR, reflecting a slight outperformance. In general, the beam steering method and $p_{vy}$ show a constant performance gain of about five times fewer errors compared to the pressure component. One can notice the relation of the steering angle to the BER by analyzing Figure 11. As expected, the cardioid-like shaped beam pattern reflects a BER with a similar cardioid shape, turned to the source direction. Although some distortions in the cardioid are noticed, as intersymbol interference also impacts the performance, the steerability is clear, where pointing to the source provides the lowest error.

The vs-ptr presents a slight degradation compared to vs-bs, but performance can be considered similar except for station 1, where all vector sensor components present low SNR (<10 dB). There is an interesting aspect of the vs-ptr performance for station 6, where even for the noisy $p_{vx}$ CIR (see Figure 7c), the high SNR of the other components is sufficient to produce a similar performance to vs-bs. However, it is clear that using such a noisy component is not advantageous, and weighting the azimuth components (for this station, $p_{vx}$ is practically zeroed) as in the vs-bsptr produces the best performance. The vs-bsptr provides a performance similar to or better than that of the vs-ptr and vs-bs, which supports the idea that using the source direction information is advantageous.

### 5.2. Changing Direction Sailing Route

After leg1, the ship moves towards the north of the vector sensor location, transmitting at station 10 and then at station 11. Stations 9, 10, and 11 are at approximately 2200 m range but onto orthogonal directions (from 105° to 20°). Thus, an inversion of the directional components’ SNR is expected and verified in Figure 8a. A BER inversion is also noticed between $p_{vx}$ and $p_{vy}$ in Figure 10. In fact, a superior performance is verified at station 11, which can be partially explained by the small DS for this station.

### 5.3. Leg2 Sailing Route

During leg2, the ship approaches the vector sensor from 2230 m range (station 11), passes through the vector sensor, and moves away up to 1260 m range (station 14). The BER performance reflects the north–south path, where $p_{vx}$ shows the highest SNR. From stations 12 to 14, the BER for both vs-bs and vs-ptr varies from 1% to 3%, whereas for the vs-bsptr it varies from 0.6% to 2%. A substantial fading effect is verified in these stations, and the DS increased from 6 to 8 ms, indicating more severe channels.

### 5.4. Summary of the Receiver Structures’ Performance

The BER analysis shown in Figure 10 provides a relation between communication performance, direction, and range. We noticed from the results that the beam steering provided a concise gain for all the tested transmission stations. Even for different CIR along the stations, we are tempted to say that the performance gain is constant, which it is not. Such results empirically indicate that the receiver with the beam steering could present a predictable performance, suggesting that this relative performance (between a vector sensor and a pressure sensor) could be found to persist, even under different experimental conditions. The vs-ptr provides a comparable performance to that of vs-bs, except for station 1. However, the slightly degraded performance can be associated with noisy components, which also results in inaccurate CIR estimations. The vs-bsptr presents the best performance, where weighting the components according to the source direction is suggested to be advantageous.

## 6. Conclusions

This paper has experimentally shown the impact of a two-axis pressure-gradient vector sensor on UWAC, highlighting the vector sensor’s steerability. In this context, the EMSO’21 field experiment has played a crucial role in allowing us to test point-to-point links in different geographic quadrants. Communication performance was quantified for several ranges and directions using individual and combined vector sensor components for comparison purposes. Among the tested receiver structures, some aspects were noted: the beam steering method is the most straightforward and computationally inexpensive compared to the passive time-reversal and the joint method; the passive time-reversal method presents a similar performance to that of beam steering, although a multichannel equalizer is needed; the joint method presents the best performance, which shows the steering advantage by weighting the azimuth components. Such structures combining vector sensor components have increased the BER performance by 2 to 10 times compared to that using only the pressure component. Moreover, a concise performance gain was verified for the tested transmission stations, even for different CIR along the stations. This result suggests that the relative performance between a vector sensor and a pressure sensor could be found under different experimental conditions.

## Data Availability

https://www.siplab.fct.ualg.pt/publications.shtml#reports. Report 02/22, accessed on 29 June 2022.
